# Evaluation of Responsiveness of Community Health Services in Urban China: A Quantitative Study in Wuhan City

**DOI:** 10.1371/journal.pone.0062923

**Published:** 2013-05-02

**Authors:** Qing Luo, Qi Wang, Zuxun Lu, Junan Liu

**Affiliations:** 1 Department of Social Medicine and Health Management, School of Public Health, Tongji Medical College, Huazhong University of Science and Technology, Wuhan, Hubei Province, PR China; 2 Department of Epidemiology and Biostatistics, School of Public Health, Tongji Medical College, Huazhong University of Science and Technology, Wuhan, Hubei Province, PR China; Tehran University of Medical Sciences, (Islamic Republic of Iran)

## Abstract

**Background:**

With the objective of the national health services systems reform to move care to the community, community health services (CHS) are becoming the gateways of the health system in China. This study aims to evaluate the levels and distributions of the responsiveness of CHS in urban China and identify the relevant features to provide the government with policy advice on the improvement of CHS responsiveness.

**Methods:**

A total of 872 face-to-face interviews were conducted in community health centers (CHCs) from 2007 to 2009. Indicators of responsiveness that were recommended by the World Health Organization were adopted, and non-conditional logistic regression analysis was performed to explore the factors associated with the levels and distributions of the responsiveness of CHS.

**Results:**

The responsiveness scored at a fairly ‘good’ level of 7.45, 7.45, and 7.46 for CHS in years 2007, 2008, and 2009, respectively. The representative responsiveness inequality indexes were 0.097, 0.101, and 0.109, respectively, indicating the moderately balanced distributions of responsiveness in these three years. During this period, the scores of responsiveness elements were highest at 7.44 to 8.34 in “dignity”, “communication”, and “social support”, while lowest at 6.76 to 7.54 in “autonomy”, “confidentiality”, and “basic amenities”. The results of the logistic regression analysis suggested that five elements (OR value), namely, “dignity” (1.414–3.345), “communication” (1.218–3.655), “basic amenities” (1.251–2.362), “prompt attention” (1.098–1.590), and “autonomy” (1.416–2.173), had significant associations with CHS responsiveness.

**Conclusions:**

The responsiveness of CHS in Wuhan City was fairly good but still requires further improvement, particularly on the working conditions of CHCs and communication skills trainings among CHS workers.

## Introduction

The framework put forward by the World Health Organization (WHO) in 2000 highlight health, responsiveness, and fairness of financing as the three main targets in the assessment of health system performance [1]. Of the three, and proposed for the first time, responsiveness refers to how well the health system meets the population expectations for the non-health enhancing aspects of the system [2]. Responsiveness requires all member states to improve responsiveness levels and reduce unfairness in the health system [3].

Responsiveness evaluation is different from patient satisfaction measurement. First, the concept of customer satisfaction was introduced into the field of marketing by Cardozo (1965). Hulka and her colleagues then initiated the conceptualization of patient satisfaction [4], [5], a mature concept with an accepted international standard called Customer Satisfaction Index (CSI). Despite being a new concept that was introduced by WHO in 2000, responsiveness is becoming a hot spot for evaluating the health service system. Second, patient satisfaction is usually evaluated on both medical and non-medical aspects, in which satisfaction with clinical nursing and medical technology is important. In the responsiveness assessment, non-medical aspects of the health system are given more focus than the medical ones. Third, patient satisfaction is a complicated mixture of expectant expectations and experiences of medical care, whereas responsiveness evaluation assesses the extent by which the health system meets the individuals’ general expectations for health services [6], [7]. Finally, the elements of responsiveness are steadier than those of patient satisfaction because responsiveness does not evaluate medical technology. Moreover, health service responsiveness is recognized as one of the basic human rights. The International Covenant on Economic, Social, and Cultural Rights (ICESCR) contains “the right to health”. Mann likewise advocated that protecting human rights is synergistic with improving public health [8]. Particularly, the elements of “dignity” and “confidentiality” best reflect human rights. Similarly, WHO stated that “without health, other rights have little meaning”. Thus, promoting human health is a prime goal of human rights. Considering the interaction between the health system and residents as well as the importance of the basic human rights maintained by the health system, WHO recommends responsiveness as one of the three main goals [2], [9], [10].

According to a survey conducted by WHO, health system responsiveness significantly vary among nations with diverse cultures, economics, and politics [11]. However, almost all countries are similar in that enhancement of responsiveness induced a profound effect on encouraging patients to seek health care from health agencies to improve their health status [7]. Although the results of the WHO survey on 191 countries demonstrated that responsiveness levels are generally higher in countries with higher individual health expenditures, several countries with health expenditures that are two or three times those of others obtained similar responsiveness [12]. An investigation in Taiwan likewise showed that various people concerned in the health system should be assessed during responsiveness comparisons, as this may present different weighted coefficients of responsiveness due to the diverse national cultures [13].

Since 1997, CHS have been greatly developed in China. In 2011, the MOH report revealed that the number of CHCs reached 32,550, and that of patient visits reached 400,000,000, increasing by 27.6% compared with that in 2010 [14]. A new round of medical reform has been initiated in China since 2009, in which CHS improvement was deemed of particular importance. One of the main goals of the new reform was to move basic medical care to the community level (i.e., CHS). However, as CHS is at the preliminary stage in China, many of its aspects required improvement. Studies on responsiveness, different from those of patient satisfaction, revealed several deep-seated problems in the development of CHS and would benefit the sustainable development of CHS.

Since 1997, Wuhan, located in the middle China, has established CHS networks to provide basic medical and public health services for over nine million inhabitants. This research aims to evaluate the levels and distributions of CHS responsiveness through field surveys, as well as to identify the factors related to the internal structure of responsiveness, to propose policy advices for government agencies on improving CHS responsiveness.

## Methods

### Ethics Statement

This study and its consent procedure were approved by the Ethics Committee of Tongji Medical College, Huazhong University of Science and Technology. All subjects were given free choice of receiving or rejecting the interview, and verbal consent was obtained from each participant prior to the study. All questionnaires were anonymously filled out by the participants.

### Research Design

**Figure 1:**
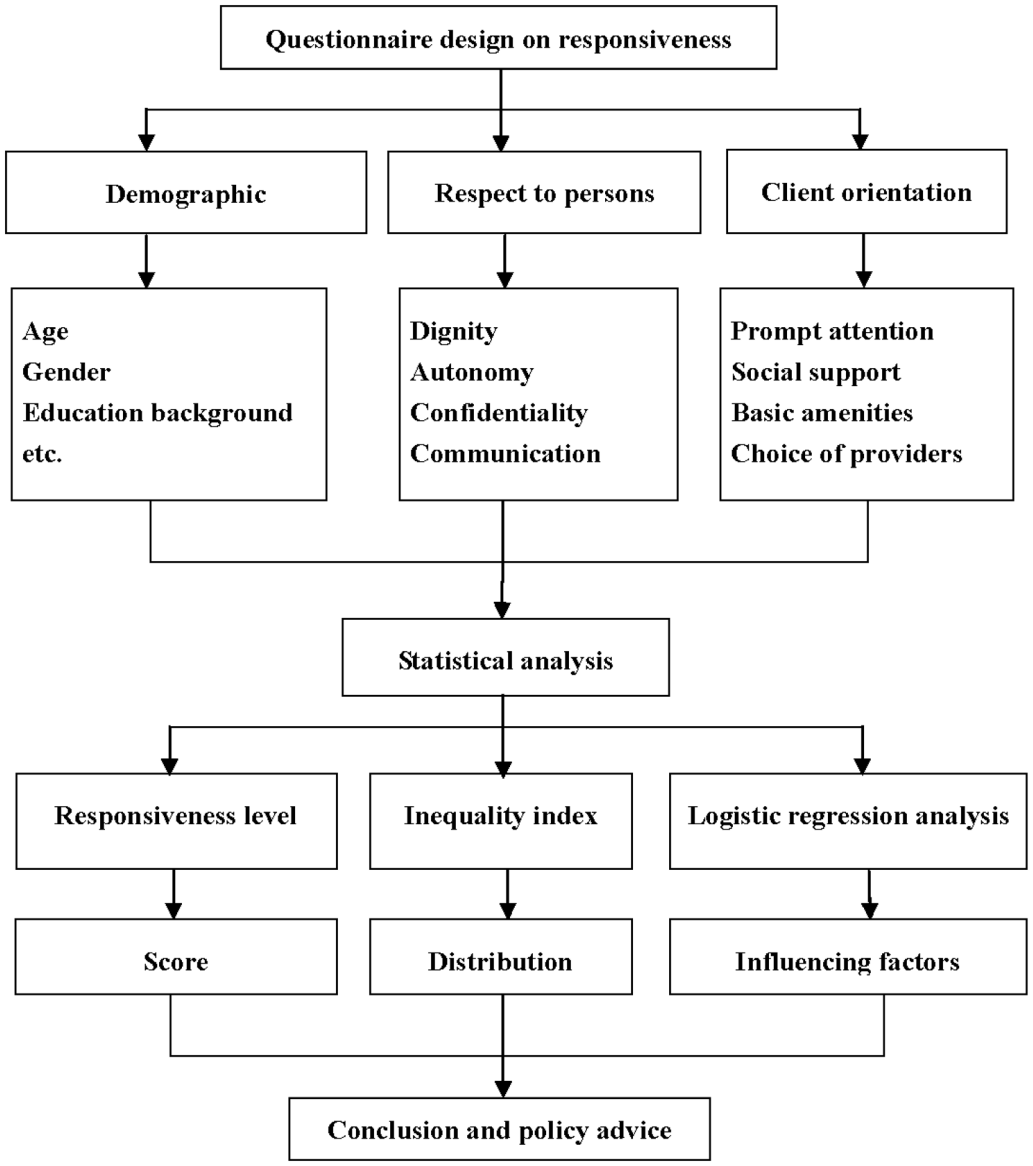
The framework of responsiveness evaluation in Wuhan community health services (CHS).

Figure 1 illustrates the research framework. Five representative communities were selected for the study. The selected CHCs are non-profit and provide services that included basic medical care, preventive care, and health education to 20,000 to 30,000 residents.

### Questionnaire Design

In 2000, WHO defined responsiveness as comprising “respects to persons” and “client orientation”. “Respects to persons” contains three elements, which are “dignity”, “autonomy”, and “confidentiality”. The 4^th^ element, “communication”, was recommended and added in 2001. “Client orientation” includes four elements, which are “prompt attention”, “social support”, “quality of basic equipment”, and “choices of providers” [15]. In designing the questionnaires for the responsiveness assessment, using a five-point Likert-type scale is deemed more reasonable than using a four-point Likert-type scale [16].

In this research, the responsiveness questionnaire was designed based on the WHO instrument of health system responsiveness in 2001. Characteristics of Wuhan CHS were likewise considered. The questionnaire comprised eight items on the personal information of residents (age, gender, education background, etc.) and 12 items on CHS responsiveness, covering dignity, autonomy, confidentiality, communication, prompt attention, social support, basic amenities, and choices of providers (Figure 1). [2], [17] The items were measured on a 5-point scale ranging from 1 (very poor) to 5 (very good). The questionnaire’s Cronbach’s alpha coefficient was 0.825, suggesting that the instrument had a good internal consistency.

### Data Collection

In this study, five CHCs were selected. These CHCs are among the centers that provided the earliest community health services in Wuhan City; hence, the residents are familiar with them. These CHCs provide medical services to average life communities rather than functional ones; hence the served population with similar characteristics to that of Wuhan residents can be regarded as a representative of these communities. Three of the five CHCs are stated-owned and the other two are collective-owned, which inflects the CHC status in Wuhan. The basic survey mode was a face-to-face interview conducted by professionally trained investigators with the residents using the self-designed questionnaire at the five CHCs in July 2007, 2008, and 2009. Under the investigators’ guidance, the respondents completed the questionnaire themselves. For those who could not independently complete the questionnaire (for reasons such as illiteracy), investigators filled out the form with the respondent’s answers. For quality control procedures, all completed questionnaires were immediately collected after the investigators carefully checked the responses and corrected any mistakes. A total of 901 questionnaires were sent out, and 872 valid ones were returned (301 in 2007, 271 in 2008, and 300 in 2009). The response rate was 96.8%. All subjects provided their informed consent prior to the investigation. Epidata3.1 was used to manage all the double-entry data. Statistical analysis was performed using SPSS version 17.0.

### Statistical Analysis

#### Scores of responsiveness levels

The WHO-recommended formula (shown below) was used to calculate the responsiveness scores, which are equal to the sum of the weighted scores of the eight elements, such as dignity, autonomy, confidentiality, communication, prompt attention, social support, basic amenities, and choices of providers. In the formula, S and W denote the representative score and weight coefficient for each element, respectively. The corresponding weight coefficients for each of the eight elements mentioned above were 0.125, 0.125, 0.125, 0.125, 0.20, 0.15, 0.10, and 0.05, respectively [1].
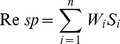



The scores of the eight elements were calculated according to the formula below, in which S, Xi, Ni, and N represent the average score of each element, responsiveness score of items contained in each element, number of respondents who correspondingly obtained the same score, and the total number of respondents, respectively. In the results, scores of 1–2, 3–4, 5–6, 7–8, and 9–10 indicate very poor, poor, average, good, and very good responsiveness, respectively.
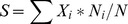



#### Responsiveness distribution

The inequality index recommended by WHO was calculated to determine the CHS responsiveness distributions [18]. The parameter was expressed as the exponent of individual**-**mean difference (IMD) ranging from 0 to 1, where 1 indicates the most unbalanced distribution and 0 indicates the most balanced one.

The formula is shown below, where Yi represents the responsiveness score (0–10) of each respondent, µ is the mean of the responsiveness scores of the respondents, and n is the sample size. The WHO-recommended values of α and β are 2 and 1, respectively.
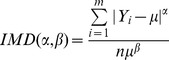



#### Non-conditional logistic regression analysis

Stepwise non-conditional logistic regression was performed to analyze the relationship between the eight mentioned elements and responsiveness, where responsiveness that was deemed very poor, poor, or average was assigned the value of 0, and those considered good or very good were assigned the value of 1. The critical thresholds of the variables entering in and rejected from the models was *P* = 0.05 and *P* = 0.10, respectively. The level of statistical significance was set at *P*<0.05.

## Results

### Demographic Characteristics of the Respondents

Table 1 summarizes the basic demographic characteristics of the survey respondents during the study period. Among the respondents, females dominated (62.8%, 59.0%, and 62.7%), and about half (49.5%, 43.5%, and 41.7%) aged between 30–59 years, whereas those above 60 years old accounted for 23% to 32%. Most respondents (81.7%, 79.6%, and 69.0%) were married and about half (55.1%, 51.3%, and 51.0%) of respondents were employed. In terms of educational background, about half (55.1%, 51.3%, and 51.0%) of the respondents received high school education. In addition, 70% to 86% of respondents received a monthly income of less than RMB 2000. Over 80% of the respondents had medical costs below 3000 RMB per year. Medical insurances cover more than 65%.

**Table 1:** Demographic characteristics of the respondents (n, %).

Variables	Groups	2007	2008	2009
Gender	Male	112 (37.2)	111 (41.0)	112 (37.3)
	Female	189 (62.8)	160 (59.0)	188 (62.7)
Age (yrs.)	15–29	80 (26.6)	64 (23.6)	93 (31.0)
	30–59	149 (49.5)	118 (43.5)	125 (41.7)
	60 and above	72 (23.9)	89 (32.8)	82 (27.3)
Marital status	Unmarried	49 (16.3)	44 (16.1)	73 (24.3)
	Married	246 (81.7)	218 (79.6)	207 (69.0)
	Others	6 (2.0)	9 (3.3)	20 (6.7)
Education background	Illiteracy	18(6.0)	14(5.1)	16(5.3)
	Primary and junior high school	82 (27.2)	102(37.2)	111 (37.0)
	Senior high school	104 (34.6)	85 (31.0)	97(32.3)
	College or above	97 (32.2)	70 (25.7)	76 (25.3)
Employment status	Employed	166 (55.1)	139 (51.3)	153 (51.0)
	Unemployed	135 (44.9)	132 (48.7)	147 (49.0)
Monthly income (RMB)	0–1999	234 (77.7)	235 (86.7)	223 (74.3)
	2000–3999	56 (18.6)	30 (11.1)	60 (20.0)
	4000 and above	11 (3.7)	6 (2.2)	17 (5.7)
Medical cost (RMB per year)	0–999	129 (42.9)	141 (52.1)	129 (43.0)
	1000–2999	144 (47.9)	115 (42.4)	121 (40.3)
	3000 and above	28 (9.3)	15 (5.5)	50 (16.7)
Insurance	Self-payment	105 (34.9)	62 (22.9)	71 (23.7)
	Medical insurance	196 (65.1)	209 (77.1)	229 (76.3)

### Responsiveness Level Scores

The responsiveness scores of the eight elements during the study period are shown in Table 2. In the three years, total responsiveness scores of Wuhan CHCs were 7.45, 7.45, and 7.46, suggesting a fairly good and steady, but lack upward momentum CHS responsiveness. Of the eight elements, “dignity”, “communication”, and “social support” had the largest mean scores (ranged from 7.44 to 8.34), while “autonomy”, “confidentiality”, and “basic amenities” had the lowest (ranged from 6.76 to 7.54).

**Table 2:** Mean responsiveness scores of Wuhan CHCs.

Elements	2007	2008	2009
Dignity	7.44	7.57	7.52
Autonomy	7.42	7.34	7.34
Confidentiality	7.02	7.28	7.54
Communication	7.69	7.65	7.48
Prompt attention	7.70	7.58	7.40
Social support	8.00	8.02	8.34
Basic amenities	6.90	6.76	6.88
Choices of providers	7.52	7.74	7.62
Total	7.45	7.45	7.46

### Responsiveness Distribution

Inequality index was used to determine the responsiveness distribution, the results of which are shown in Table 3. To describe the better responsiveness distributions, we classified “very poor” and “poor” as “low” responsiveness level, “average” as the “middle”, and “good” and “very good” as “high”. The means of responsiveness inequality indexes were 0.097, 0.101, and 0.109, for the years 2007, 2008, and 2009, respectively, suggesting that responsiveness distributions were generally balanced in Wuhan CHS.

**Table 3:** Responsiveness distributions among the eight elements of CHC responsiveness (n, %).

Elements	Year	Low	Middle	High	Responsiveness inequality index
Dignity	2007	0 (0)	60 (19.93)	241 (80.07)	0.018
	2008	11 (4.06)	35 (12.92)	225 (83.02)	0.223
	2009	8 (2.67)	54 (18.00)	238 (79.33)	0.193
Autonomy	2007	12 (3.98)	98 (32.56)	191 (63.46)	0.360
	2008	27 (9.96)	72 (26.57)	172 (63.47)	0.386
	2009	12 (4.00)	106 (35.33)	182 (60.66)	0.285
Confidentiality	2007	1 (0.3)	160 (53.2)	140 (46.52)	0.206
	2008	13 (4.80)	94 (34.69)	164 (60.52)	0.284
	2009	1 (0.33)	93 (31.00)	216 (68.67)	0.192
Communication	2007	7 (2.32)	47 (15.61)	247 (82.07)	0.260
	2008	19 (7.01)	34 (12.55)	218 (80.44)	0.241
	2009	13 (4.33)	55 (18.33)	232 (77.33)	0.205
Prompt attention	2007	19 (6.31)	68 (22.59)	214 (71.10)	0.361
	2008	21 (7.75)	58 (21.40)	192 (70.85)	0.370
	2009	29 (9.67)	57 (19.00)	214 (71.33)	0.349
Basic amenities	2007	13 (4.32)	154 (51.16)	134 (44.51)	0.251
	2008	15 (5.54)	151 (55.72)	105 (38.75)	0.273
	2009	34 (11.33)	128 (42.67)	138 (46.00)	0.223
Social support	2007	1 (0.33)	64 (21.26)	236 (78.40)	0.311
	2008	7 (2.58)	52 (19.19)	212 (78.23)	0.257
	2009	2 (0.67)	43 (14.33)	255 (85.00)	0.438
Choices of providers	2007	14 (4.64)	53 (17.61)	234 (77.75)	0.306
	2008	23 (8.49)	19 (7.01)	229 (74.50)	0.258
	2009	18 (6.00)	35 (11.67)	247 (82.33)	0.264

Among the eight elements, all responsiveness inequality indexes were below 0.5 during the three years, indicating relatively balanced responsiveness distributions. According to our results, the index of dignity is closer to 0 than the corresponding values of the others, suggesting the most balanced distribution of “dignity” responsiveness. However, particular attention should likewise focus on the changes of responsiveness distribution indexes for “autonomy”, “prompt attention”, and “social support”.

### Demographic Characteristics Associated with CHS Responsiveness

Table 4 displays the demographic characteristics associated with CHS responsiveness. The difference between the males and females was significant in 2007 (*P*<0.01); the responsiveness levels grouped by medical expenditure exhibited significant difference in 2008 (*P*<0.05); and variables such as gender (*P*<0.05), marital status (*P*<0.01), age (*P*<0.01), educational background (*P*<0.01), occupation (*P*<0.01), monthly income (*P*<0.05), and medical expenditure (*P*<0.05) were associated to CHS responsiveness in 2009.

**Table 4:** Responsiveness scores of CHCs grouped by demographic characteristics.

Variables	Groups	2007		2008		2009	
		Scores	F	Scores	F	Scores	F
Sex	Male	7.16	9.219**	7.46	0.057	7.31	4.600*
	Female	7.47		7.44		7.54	
Marital status	Married	7.43	0.453	7.47	1.263	7.11	10.005**
	Unmarried	7.33		7.46		7.53	
	Others	7.56		7.00		7.98	
Age (yrs.)	15–29	7.41	0.518	7.45	0.003	7.25	12.273**
	30–59	7.36		7.45		7.26	
	60 and above	7.27		7.44		7.78	
Education background	Illiteracy	7.60	0.888	7.08	2.023	7.66	7.230**
	Primary and junior high school	7.41		7.36		7.69	
	Senior high school	7.29		7.60		7.45	
	College or above	7.33		7.46		7.09	
Employment status	Employed	7.27	3.192	7.45	0.000	7.36	14.692**
	Unemployed	7.44		7.45		7.64	
Insurance type	Self- payment	7.41	0.770	7.48	0.137	7.31	2.491
	Medical insurance	7.32		7.44		7.50	
Monthly income(RMB)	0–1999	7.38	0.689	7.44	0.195	7.55	4.089*
	2000–3999	7.29		7.53		7.23	
	4000 and above	7.12		7.31		7.14	
Medical cost(RMB per year)	0–999	7.42	1.871	7.29	3.666*	7.40	3.618*
	1000–2999	7.21		7.59		7.43	
	3000 and above	7.26		7.82		7.70	

Note: Statistical analysis revealed significantly different responsiveness scores with a significance of *P<0.05 or **P<0.01.

### Non-conditional Logistic Regression Model of CHS Responsiveness

Table 5 shows the results of the non-conditional logistic regression analysis on the relationship of CHS responsiveness with the eight mentioned elements. The three regression models fit well and were statistically significant (2007: *χ^2^* = 77.812, *P*<0.001; 2008: *χ^2^* = 142.178, *P*<0.001; and 2009: *χ^2^* = 89.944, *P*<0.001). In these models, elements of “dignity” (2007 and 2008), “communication” (2007 and 2008), “basic amenities” (2007 and 2008), “prompt attention” (2009), and “autonomy” (2009) influenced CHS responsiveness. The most significant impact on responsiveness was produced by “basic amenities” (OR = 2.362) in 2007, “communication” (OR = 3.655) in 2008, and “autonomy” (OR = 2.173) in 2009.

**Table 5:** Results of non-conditional logistic regression analysis for the three years.

Variables	2007			2008			2009		
	β	OR	95%CI	β	OR	95%CI	β	OR	95%CI
Dignity	0.632	1.882*	1.104–3.209	1.207	3.345**	1.873–5.972	0.344	1.411	0.902–2.206
Autonomy	0.348	1.416	0.749–2.677	0.436	1.546	0.833–2.870	0.776	2.173*	1.128–4.188
Communication	0.495*	1.641*	1.081–2.490	1.296	3.655**	2.173–6.147	0.197	1.218	0.816–1.817
Prompt attention	0.257	1.294	0.807–2.073	0.094	1.098	0.622–1.937	0.464	1.590*	1.019–2.481
Basic amenities	0.860	2.362**	1.254–4.451	1.251	3.494**	1.468–8.317	0.465	1.592	0.971–2.609

* P<0.05,

** P<0.01.

## Discussion

Ensuring residents’ access to the basic package of comprehensive health services is an important issue in China. Developing CHS is not only the proper solution to the problem, but may likewise optimize the performance of the national health system. WHO regards responsiveness as one of the main goals of the health system because it is a constant indicator that considers the non-medical aspects of the health system [19]. Effective responsiveness of the health system results in good compliance of healthcare providers, and patients would be happy to seek health services when they are sick [20]. Thus, evaluating CHS performance by assessing responsiveness in the health system reform is more authoritative. Furthermore, responsiveness and its tools have been widely used to evaluate the mental intervention service system, HIV testing, and counseling services, among others. Such applications demonstrate that the concept of responsiveness developed by the WHO is also applicable to other special health services [21], [22], [23].

In 2000, WHO first listed the average levels of responsiveness of the health systems of 191 member nations. Among them, China ranked 88^th^ with a responsive score of 7.33, indicating a middle-level responsiveness [12]. Our study showed that responsiveness at Wuhan CHS scored higher with 7.45 (2007), 7.45 (2008), and 7.46 (2009), demonstrating excellent constructions of CHS in Wuhan City. According to other domestic reports, Wuhan obtained CHS responsiveness scores that were higher than those of Shandong province (7.13), Shenzhen City (6.83), and Fuzhou City (4.72), but lower than that of Shanghai City (8.13) [19], [24], [25], [26]. The questionnaires utilized in these CHS responsiveness studies were likewise based on the WHO responsiveness survey. Although the characteristics of the Wuhan population are similar to those of the cities mentioned above, certain differences still exist between Wuhan and the other cities. The Shenzhen population is younger than that of Wuhan because Shenzhen is a young city. Although Wuhan and Shanghai are both mature cities in China, the proportion of old people (aged 60 years and above) to the whole population in 2009 was 22% in Shanghai, which was larger than that in Wuhan (13%). Therefore, comparison among the four cities should be more cautious. For responsiveness distributions of Wuhan CHS in 2007, 2008, and 2009, the representative inequality indexes were 0.097, 0.101, and 0.109, respectively. All these indexes had no significant difference from zero, which suggested a relatively balanced distribution of responsiveness. In other words, community medical staff have treated patients equally without discriminations. Yan Y et al.’s survey showed that 95.2% of the residents were satisfied with the overall quality of the CHS after the profit-driven nature began phasing out from Wuhan CHCs [27].

However, nearly at the same levels in three years, Wuhan CHC responsiveness lacked a force to further improve, particularly in the aspects of “confidential”, “autonomy”, and “basic amenities”. By contrast, urgent improvements are necessary in the aspects of “autonomy” and “choices of providers” in Shandong, whereas “dignity”, “prompt attention”, and “basic amenities” in Shenzhen [19], [24]. According to reports from South Africa and Brazil, the three aspects with the lowest ranking were “prompt attention”, “autonomy”, and “basic amenities”, which were similar to Wuhan except for “prompt attention”. The major reason for patients’ dissatisfaction was that the Primary Health Care (PHC) facilities could not adequately shoulder a large burden and responsibility in South Africa and Brazil [28], [29], similar to the situation of Wuhan CHS.

Compared with large-scale hospitals, CHCs have more advantages in terms of respect to patients, social support, and choices of providers. However, the latter had insufficient governmental investment, resulting in a big gap in infrastructure and environmental constructions, especially the shortage of space for protecting patient privacy. In addition, doctors who do not properly recognize patients’ autonomy deem that patients must follow the doctor arrangement in the process of medical decision-making. With the position of CHS rising gradually in the national health system, the government should exert effective efforts on CHS constructions, pay attention to environmental improvement, and heighten the construction standards. Community physicians should conduct systematic patient-centered services, provide sufficient answers to patient questions, and carefully protect patient privacy to gain their trust.

Although CHS responsiveness was associated with demographic characteristics, such as gender, age, education level, and so on, the responsiveness scores were at good levels as grouped by the variables mentioned above, which agreed with the balanced responsiveness distributions. The differences of responsiveness scores among the population subgroups were mostly caused by their diverse demands on CHS. With its increasing utilization, people would demand more from CHS; hence, responsiveness levels would be more sensitive to demographic factors. That is, the particular needs of different populations require clarification, which would be helpful to the oriented improvement of CHS responsiveness, such as in increasing the convenience for the elderly to receive medical treatment, timely requests for medical treatment of young people, and so on.

Our findings reveal that various factors are related to CHS responsiveness in Wuhan during the study period, and more variables affected CHS responsiveness in 2009 compared with the other two years. This result may be a consequence of the economic development and the new round of medical reform that began in 2009. As the overall income of urban residents increased in pace with economic growth, the per capita monthly disposable income (China yuan) were 1196.47 in 2007, 1372.70 in 2008, and 1532.09 in 2009 [30]. Residents who require high levels of CHS have correspondingly higher expectations on CHS. In the new medical reform, the government allocated more investments into CHS to promote essential drug policies and then canceled drug markup; thereby resulting in an increase in CHS utilizations among community residents and subsequent increased complaints on health services. As such, more demographic characteristics were closely associated with CHS responsiveness in 2009.

Among the eight elements of responsiveness, “dignity”, “communication”, “basic amenities”, “prompt attention”, and “autonomy” significantly affected responsiveness. This result indicates that better responsiveness can be achieved by improving these five elements. Valentine N disclosed that the most important among the elements was “prompt attention”, followed by “dignity”, and third is “communication” [31]. However, our findings demonstrated that “basic amenities”, “communication”, and “autonomy” were the most important elements of responsiveness in Wuhan CHS. In comparison with large-scale hospitals in Wuhan, CHCs were more widespread and consequently provider higher convenience for residents to seek health care. Therefore, “prompt attention” was well achieved in CHCs. For this reason, the “basic amenities” of CHCs as compared with large-scale hospitals was the main element affecting responsiveness. “Communication” likewise serves an important role in both large-scale hospitals and CHCs.

To improve CHS responsiveness, the further enhancement of software and hardware constructions of CHCs is essential. In improving software constructions, healthcare givers in CHCs should enhance the consciousness of service and communication skills with residents through different types of occupational training that does not need much economic investment. In addition, hardware constructions have the same importance, but improvements of “basic amenities”, “choices of providers”, and “prompt attention” require governmental economic investment and human resource allocation.

Our study has certain limitations. Our research adopted the WHO responsiveness and a simplified questionnaire, which were more feasible, but needed further study to consummate in the future. In addition, several researchers argue that the measurement tool of responsiveness requires substantial revision to capture other important dimensions [22]. Moreover, the sample sizes of the survey sites and subjects were smaller than expected, thereby decreasing the external applicability of the conclusions.

## References

[1] WHO (2000) Health system: Improving performance. The World Health Report 2000, Geneva.

[2] Darby C, Valentine N, Murray CJL, De Silva A (2001) World Health Organization (WHO): strategy on measuring responsiveness. GPE Discussion Paper Series: No.23, Geneva, WHO.

[3] Qu J, Li S, Wang X, Lv S (2001) World Health Organization’s strategy on the responsiveness scaling of health system. Health Economics Research 5: 9–11.

[4] Cardozo RN (1965) An experimental study of customer effort, expectation and satisfaction. Journal of Marketing Research 2: 244–249.

[5] Hulka BS, Zyzanski SJ, Cassel JC, Thompson SJ (1970) Scale for the measurement of attitudes toward physicians and primary medical care. Med Care 8: 429–436. 4920640 10.1097/00005650-197009000-00010

[6] Smith C (1992) Validation of a patient satisfaction system in the United Kingdom. Qual Assur Health Care 4: 171–177. 1391786 10.1093/oxfordjournals.intqhc.a036716

[7] De Silva A, Valentine N (2000) Measuring responsiveness: results of a key informants survey in 35 countries. GPE Discussion Paper Series: No.21 Geneva: WHO.

[8] Mann JM, Gostin L, Gruskin S, Brennan T, Lazzarini Z, et al (1994) Health and Human Rights. Health Hum Rights 1: 6–23. 10395709

[9] Jamar SD (1994) The international human right to health. South Univ Law Rev 22: 1–68. 12741379

[10] Gostin LO (2003) The domains of health responsiveness: a human rights analysis: World Health Organization. 12 p.

[11] Navarro V (2001) The new conventional wisdom: an evaluation of the WHO report, Health Systems: Improving Performance. Int J Health Serv 31: 23–33. 11271646 10.2190/3LM8-A37Q-FKJ4-TE0R

[12] Valentine NB, de Silva A, Murray CJL (2000) Estimating responsiveness lever and distribution for 191 countries: methods and results. GPE Discussion Paper Series: No.22, EIP/GPE/FAR, WHO.

[13] Hsu CC, Chen L, Hu YW, Yip W, Shu CC (2006) The dimensions of responsiveness of a health system: a Taiwanese perspective. BMC Public Health 6: 72. 16542462 10.1186/1471-2458-6-72 PMC1459139

[14] MOH (2011) The national medical services results in Jan.-Oct. Ministry of health website. Available: http://www.moh.gov.cn/mohwsbwstjxxzx/s7967/201112/53508.shtml. Accessed 2012 Sep 25.

[15] Bedirhan Üstün T, Chatterji S, Villanueva M, Bendib L, Celik C, et al.. (2001) WHO multi-country survey study on health and responsiveness 2000–2001. GPE Discussion Paper Series: No. 37 Geneva: WHO.

[16] Jiang Q, Hu S, Li G, Ying X, Liu B, et al (2002) Analysis of the responsiveness of health system in Shanghai. Chinese Health Service Management 168: 324–332.

[17] De Silva A (2000) A framework for measuring responsiveness. GPE Discussion Paper Series: No.32 Geneva: WHO.

[18] Murray CJL, Evans DB (2003) Health systems performance assessment: debates, methods and empiricism. Geneva: WHO.

[19] Yang D, Li J, Sun Y, Lu Z (2005) Analysis on responsiveness assessment and influence factors of community health service in Shenzhen. Chinese General Practice 8: 359–362.

[20] Murphy-Cullen CL, Larsen LC (1984) Interaction between the socio-demographic variables of physicians and their patients: Its impact upon patient satisfaction. Soc Sci Med 19: 163–166. 6474232 10.1016/0277-9536(84)90283-1

[21] Peng D, Li X, Zhang Q, Zhu C, Zhang J, et al (2011) Responsiveness evaluation of mental intervention services system in Wenchuan earthquake area. Chin J Prev Med 45: 158–162. 21426798

[22] Njeru MK, Blystad A, Nyamongo IK, Fylkesnes K (2009) A critical assessment of the WHO responsiveness tool: lessons from voluntary HIV testing and counseling services in Kenya. BMC Health Serv Res 9: 243. 20028540 10.1186/1472-6963-9-243 PMC2811110

[23] Forouzan AS, Ghazinour M, Dejman M, Rafeiey H, Sebastian M (2011) Testing the WHO responsiveness concept in the Iranian mental healthcare system: a qualitative study of service users. BMC Health Serv Res 11: 325. 22115499 10.1186/1472-6963-11-325 PMC3280196

[24] Ma Q, Yin W, Huang D, Meng M, Li D (2009) Study on patients’ responsiveness community health service of three cities in Shangdong Province. Chinese Primary Health Care 23: 26–29.

[25] Wang X, Zheng Z, Wang H, Jiang Q (2008) Fuzzy synthetic discrimination on the responsiveness of community health service in Fuzhou. Chinese Primary Health Care 22: 18–20.

[26] Zhang J, Yao Y, Li C (2007) Analysis on responsiveness assessment of community health service in Zhabei District of Shanghai. Chinese Primary Health Care 21: 28–30.

[27] Yan Y, Liu Y, Liu X, Yang N, Xia J (2009) Study on awareness rate and satisfaction to community health service among urban residents in Wuhan City. Chinese Journal of Social Medicine 26: 184–186.

[28] Peltzer K (2009) Patient experiences and health system responsiveness in South Africa. BMC Health Serv Res 9: 117. 19602290 10.1186/1472-6963-9-117 PMC2716320

[29] Gouveia GC, Souza WV, Luna CF, Souza-Júnior PR, Szwarcwald CL (2005) Health care users' satisfaction in Brazil, 2003. Cad Saude Publica 21: 109–18. 16463002 10.1590/s0102-311x2005000700012

[30] Satistics Bureau of Wuhan Municipality (2010) Wuhan statistical. yearbook-2010: Page264.

[31] Valentine N, Darby C, Bonsel GJ (2008) Which aspects of non-clinical quality of care are most important? Results from WHO's general population surveys of “health systems responsiveness” in 41 countries. Soc Sci Med 66: 1939–1950. 18313822 10.1016/j.socscimed.2007.12.002

